# Case of a Needle Entering the Right Breast, and Finally Lodging in the Heart, Causing Death

**Published:** 1843-05-27

**Authors:** B. F. Leaming

**Affiliations:** Physician to the Philadelphia Dispensary


### CASE OF A NEEDLE ENTERING THE RIGHT BREAST,

*And finally lodging in the heart, causing death.*

By B. F. Leaming, M. D.

Physician to the Philadelphia Dispensary.

On the 4th of August, 1842, a seamstress, in good health, eighteen years of age, came to my office, requesting me to remove a needle which she supposed had penetrated the right breast. Two days before, while in the act of bending suddenly forwards, she had struck a table, and driven a needle, which was sticking in her dress, forcibly into the breast. She was not quite certain that the needle was still there; sometimes she thought she could feel it, at other times she was sure it was not there. On examination I found a puncture about an inch from the nipple, a little below the margin of the areola, and on the side towards the sternum. Her breast was rather large; it could be pressed firmly in any direction without causing pain; she had no cough, and could take full inspirations without inconvenience.

On the 8th of September she had pleurisy of the right side; she had not been exposed to cold, but the pain and cough and difficulty of breathing had come on suddenly after she had stooped to pick something from the floor. The inflammation yielded to the usual antiphlogistic remedies, and in three weeks she regained her usual health.

On the 13th of February, 1843, she had a slight attack of pneumonia of the lower anterior part of the right lung; she had also bronchitis on both sides. She recovered in a week from the acute symptoms, though afterwards she was always affected with a troublesome cough.

On the 10th of March she had spasms of the diaphragm; they continued for three or four days, intermitting occasionally for a few hours. The inconvenience was not sufficient to confine her to the chamber. Until this time the pulse had been regular, and always corresponded to the degree of fever present.

On the 26th of March she had obstinate vomiting, not accompanied by tenderness at the epigastrium, nor by thirst for cold drinks, nor by constipation, nor by any cerebral symptoms whatever. The vomiting continued four days; it was always increased by drinks, though the efforts frequently persisted while the stomach was quite empty. During the intervals her pulse was 80, feeble and regular.

On the 5th of April she had pain in the heart. During my attendance at the previous illness of the patient, my prescriptions had been so much neglected that I declined visiting her any more; therefore I did not examine her symptoms particularly when called on at this time, (5th of April,) but requested the friends of the patient to seek advice elsewhere.

On the 8th of April she was visited by Dr. Franklin Bache, who detected the existence of pericarditis, accompanied by a very irregular and feeble pulse. From inattention on the part of the patient to Dr. Bache's prescriptions, his visits were continued only four days. She was then attended by Drs. Watson and Arrott, who found her in a very prostrate condition, with irregular pulse, and sometimes so feeble that it could scarcely be felt; she had also the usual symptoms of pneumonia.

On the 15th of April she had œdema of the feet; and two days afterwards her face was somewhat swollen.

She died on the 27th of April, nearly nine months after the needle had entered her body.

On examination, after death, the right lung was found adherent throughout to the costal pleura; both lungs were congested, and the bronchia inflamed; at the posterior inferior part of each lung there was some hepatization. The pericardium contained more than a quart of fluid blood; its surface was covered with deeply reddened lymph; some of the recesses formed by small folds of the pericardium, or by adhesions near the upper part of the auricles, were filled with grumous blood; there were adhesions at the under surface of the heart, particularly under the right ventricle, and in a line parallel with, and half an inch from the septum. The heart was small and flaccid, containing only small coagula. On opening the left ventricle the point of a needle was seen protruding a quarter of an inch towards the middle of the cavity. The needle had passed at the under surface of the heart about three-quarters of an inch from its apex, and half an inch from the septum, through the wall of the right ventricle, through a columna carnea, through the septum and into the left ventricle. It was fifteen lines in length; its head, somewhat incrusted, was imbedded in the wall of the right ventricle, just under the surface of the pericardium. The orifice made by the needle seemed to have been completely closed by coagulable lymph; but it would be very easy for a small channel to escape detection in such a mass. The internal membranes of the heart were smooth and shining, without any marks of inflammation; all the large vessels were in excellent condition.

*Remarks.*

Although the patient could never point to the situation of the needle, and was always unconscious of its presence in any particular part, yet, during my attendance, I never doubted that it was the cause of all her ailments. I presume it first lodged obliquely in the intercostal muscles, or was made to do so by her own attempts to discover its situation; it then passed through the pleura, and probably a portion of the lung, reaching the floor of the diaphragm; it then seems to have moved near the œsophagus, irritating some filaments of the par vagum; and finally, it reached the heart.

The blood in the pericardium may have derived either from the cavities of the heart, or from the inflamed adhesions, or from both. In the first case, we may suppose that every contraction of the heart separated the muscular fibres sufficiently to allow the blood to pass along the sides of the needle; and the separation of the fibres of the walls of the right ventricle would be greatly favoured by the *working* of the needle, caused by its extremities being subjected to differently acting forces. It is true no channel through the adhesions of the pericardium could be detected; but in the relaxed condition of the heart a small channel through coagulable lymph would necessarily escape detection. From whatever source it derived, the blood was of good appearance, resembling that drawn from the arm of a moderately anemic person; it therefore could not have been much diluted by serum from the inflamed pericardium.

